# Monocular blur impairs heading judgements from optic flow

**DOI:** 10.1177/20416695251317148

**Published:** 2025-02-26

**Authors:** William E. A. Sheppard, Rachel O. Coats, Richard M. Wilkie, Rigmor C. Baraas

**Affiliations:** School of Psychology, Faculty of Medicine and Health, 4468 University of Leeds, UK; National Centre for Optics, Vision and Eye Care, Faculty of Health and Social Sciences, 11310 University of South-Eastern Norway, Kongsberg, Norway

**Keywords:** perception, action, cognition, optic flow, heading, blur

## Abstract

Monocular blur sometimes impairs locomotion; however, it is not always clear when this will happen. Optic flow (the apparent motion of scene texture elements that occurs during self-motion) provides powerful signals about the direction of travel. Here, we test whether monocular blur impairs heading perception from optic flow compared to full vision under various levels of optic flow degradation. Participants (*N* = 52, mean age = 30 years) completed contrast sensitivity, visual acuity, and heading perception tasks with rich or degraded optic flow, with or without monocular blur (0.4 logMAR Bangerter filter over the non-dominant eye, full vision in the dominant eye). Heading perception was assessed using a browser-based task where the participants viewed a 3-second video consistent with self-motion over a textured ground plane (moving towards the horizon at an offset heading ranging from −20 to +20°) and identified the point on the horizon towards which they were travelling. The measures of each participant's performance were the absolute and directional angular error between the heading offset and their response. Monocular blur and degraded flow were associated with an increase in absolute heading error and a larger underestimation of heading angle, with the worst performance observed when monocular blur and degraded flow were combined. These results suggest that the impact of monocular blur on heading perception becomes apparent only when optic flow signals are weak (e.g., night-time driving). These findings support the theory that monocular blur and the richness of visual information interact to produce deficits in heading perception.

## How to cite this article

Sheppard, W. E. A., Coats, R. O., Baraas, R. C., & Wilkie, R. M. (2025). Monocular blur impairs heading judgements from optic flow. *i–Perception*, 16(0), 1–20. https://doi.org./10.1177/20416695251317148

The control of locomotion (travelling on foot or driving a car) is an essential functional capability for most humans. Retaining mobility is critical for maintaining quality of life (QoL) throughout older age, but visual-motor impairments can have a significant negative impact on this function (Owsley, 2002). Here, we use ‘mobility’ to mean self-propelled movement around the environment (e.g., walking, driving, and wheel-chair use). The active sampling of visual information from the world supports locomotion (Mole et al., 2021), with the flexible, weighted combination of multiple cues supporting robust steering control even when some visual information has become degraded (for example, driving at night: Wilkie & Wann, 2002). Despite these robust mechanisms, research has demonstrated that visual blur impairs locomotion (Elliott et al., 1997, 2000), particularly with low levels of illumination (Elliott et al., 1996). The conditions maximising the impact of visual blur on mobility remain unclear despite blur being an expected visual deficit within the general population (McKibbin et al., 2018).

To successfully maintain a straight-line trajectory in the presence of external perturbations (e.g., buffeting by wind), an individual must detect the direction of travel (‘heading’) to ensure it aligns with their goal and to avoid collisions. Several informational variables could provide informative heading signals when moving through the environment, which may become degraded with increased blur. Optic flow (the apparent motion of texture elements during self-motion) is a crucial perceptual signal for determining the direction of travel, whereby, an individual wishing to travel towards a point would align the ‘focus of expansion’, the point from which the flow field appears to originate, with the target and head towards that point (Gibson, 1958). Optic flow signals due to self-movement may also be parsed out to determine the direction of travel of other objects in the scene (Warren & Rushton, 2008a,b). Studies of heading perception have demonstrated sensitivity of approximately 2° to 4° depending on optic flow properties (Bruggeman & Warren, 2009; Cutting et al., 1992). Humans are sensitive to changes in optic flow; reducing optic flow quality and quantity can impair heading judgments. Kountouriotis and Wilkie (2013) used computer displays to produce dot flow patterns (a set of white dots on a black background moving in a manner consistent with forward motion over a ground plane). The researchers could degrade the dot flow by reducing the luminance of the dots by up to 75% or reducing the number of dots across the whole display (from a maximum of 3,645 to a minimum of 45). Reducing the dot luminance or the dot quantity in isolation was insufficient to significantly impair heading judgements. However, when combined, the most degraded displays produced conditions where the magnitude of heading errors became potentially unsafe (i.e., exceeded 6°). This research demonstrates considerable sensitivity to optic flow, but that sufficiently degrading optic flow can impair heading judgements.

The present question is whether visual blur (and its interaction with the richness of visual information), commonly experienced by those with visual impairments, can degrade the motion signals available from optic flow (and hence lead to impaired heading perception). Visual blur can occur monocularly or binocularly, but presently, in the UK, monocular visual impairments are most common: an estimated 10.3% of adults report a mild or worse (greater than 0.3 logMAR) monocular visual impairment compared with 2.6% who report binocular impairment (McKibbin et al., 2018). The most commonly diagnosed causes of monocular visual impairments are cataracts (15.1%), amblyopia (12.9%), and uncorrected refractive error (12.8%) (McKibbin et al., 2018), all of which are associated with blurring of vision in one eye (Hopkins et al., 2020; Liu et al., 2017; Webber & Wood, 2005). In cases where individuals have bilateral cataracts, in England and Wales, it is typical for patients to have their cataracts removed in two separate surgical sessions (Buchan et al., 2020; Dickman et al., 2022). This separation has provided valuable data for testing whether removing visual blur from one or both eyes improves locomotor control, specifically regarding vehicle control and driving function.

In a driving simulator study, the removal of both cataracts (Second-Eye Surgery; SES) reduced crashes/near-crashes by 47%, compared to the removal of the first cataract only (First-Eye Surgery; FES) (Meuleners et al., 2021). This change in performance was not associated with a change in visual acuity (VA) or stereopsis and was only weakly associated with contrast sensitivity (CS). On average, CS improved by 0.1 log units from pre-surgery to post-SES, accompanied by a relatively small (3.1%) reduction in crashes/near-crashes. Whilst crashes/near-crashes would seem to be an appropriate method for considering the real-world impact of cataracts on driving, these measures do not necessarily help to explain the underlying cause of the impairments in vehicle control (crashes/near-crashes were identified based on the subjective categorisation of rapid evasive manoeuvres such as braking or swerving in response to experimentally introduced hazards). Whilst some parametric variables of visual-motor control were examined (lane excursions, speed variation, and speed limit compliance), only speed limit compliance changed significantly after FES/SES (time spent > 10 km/h over the speed limit reduced after FES/SES). One possible interpretation of the lack of any reliable change in lane-keeping post-FES/SES is that optic flow processing was not impaired by cataract blur or improved by cataract surgery. However, a problem with this interpretation is that the visual environments presented were rich city scenes with high-contrast black roads with white road markings (Meuleners et al., 2021), which, despite the degradation of the participants’ ability to interpret optic flow, may well have provided sufficient information to control lane-keeping. Previous research has shown that demarcated road edges provide powerful steering control signals, and whilst optic flow can still influence steering behaviour, there seems to be some independence in the information provided by these two sources (Kountouriotis et al., 2012). Whilst the speed-related measures reported by Meuleners et al. (2021) could be related to optic flow processing (the finding that speed limit compliance improved after surgery could be due to improved sensitivity to vection signals available from optic flow), the lack of change in speed variation post-FES/SES suggests that this may be due to an ‘improved ability to read and recognise the speed limit signs’ (Meuleners et al., 2021). These points further highlight the difficulties in interpreting changes in driving behaviours when examining visually rich driving scenarios populated with an array of informational variables where an individual may select one of many behavioural responses.

The present study, therefore, aims to test the impact and interaction of monocular blur and a degraded flow field on a heading perception task. With full vision and monocular blur, the participants view moving scenes consistent with the observer travelling across a textured ground plane towards a point on the horizon. After the motion ends, the participant clicks on the point on the horizon towards which they judged they were travelling. We predict that monocular blur will be associated with increased error in heading judgements. The absence of additional visual information (e.g., demarcated road edges, pedestrians, or road signs) will ensure that any variation in performance is due to the visual conditions rather than higher-level ‘cognitive’ factors. The quality of the flow field is manipulated independently of the visual condition by altering the texture of the ground plane, which tests the prediction that degrading the flow field impairs heading perception and that the size of this effect increases as the flow field is further degraded. We also predict that the impairment in heading perception associated with monocular blur (relative to full vision) will increase as the flow field is further degraded. To further probe the impact of monocular blur and degrading the flow field on heading perception, we will also test the association of these factors with the directional bias in the participants’ responses across the range of heading offsets. To allow comparisons with previous research, the participant's CS and VA will be measured to establish whether visual blur's effects depend on changes to these measures.

## Methods

### Participants

An opportunity sample of 60 individuals recruited in the UK participated in the present study. Of these, the researchers excluded 11 participants, leaving a final sample of 49. Four participants were excluded due to having ocular comorbidities, three due to persistently poor performance in the training phase, one due to an error in coding at the point of data collection and three were removed due to moving from good performance on one level (70% plus) to 0% correct on the next level. A curve could, therefore, not be fitted to these data, and they were excluded from the analysis (as per Sheppard et al., 2024).

The ages of the participants ranged from 20 to 69 (*mean* age = 29.98 years, *SD* = 13.78) years old, and 75.51% of the sample was female. 53.06% of the participants reported ‘A’ or ‘AS’ level as their highest qualification, 30.61% undergraduate degree, 10.20% postgraduate qualification (Masters or PhD), and 6.12% vocational qualification (GNVQ or BTEC). Most participants (97.96%) reported using the mouse with their right hand. All participants self-reported normal or corrected-to-normal vision.

Participants were required to be over the age of 18 and to understand written English. The University of Leeds School of Psychology Ethics Committee granted ethical approval on 02/11/2021 (ethics reference number: PSYC-395).

### Design

Participants completed three tasks (CS, then VA, then heading perception) in two visual conditions (no blur, or monocular blur in the non-dominant eye) in a within-subjects experimental design. Each participant completed the three tasks in the same order; however, the order of the visual conditions was counterbalanced between participants. In the heading perception task, the order of the optic flow conditions and heading offsets was pseudo-randomised (interleaved, such that participants never did the same trial twice in a row) so that the participants would complete all combinations of these two factors before completing them again in a different pseudorandom order.

### Procedure

For practical reasons, three research assistants collected the data using different laptops (see Supplemental Material 1 for details). Testing sessions were organised at the researchers’ homes because the University laboratories were closed due to COVID-19-induced restrictions. Each participant was presented with an information sheet and was free to ask any questions before signing a digital consent form. The participants completed the demographics questionnaire followed by the eye dominance test (described in the Apparatus section). Having successfully established eye dominance, the participant was fitted with the first pair of glasses (clear lenses over both eyes or a clear lens over the dominant eye and blurred one over the other) depending on the visual condition. The participant completed the CS, VA, and heading perception tasks in that order. Once complete, the participant removed the glasses, and the researchers offered them a rest break. When ready to continue, the participant was fitted with the second pair of glasses and repeated the tasks. The participant then removed the glasses and was debriefed.

### Apparatus

In the monocular blur condition, all participants wore 0.4 logMAR Bangerter filters over their non-dominant eyes. The researcher established the participants’ eye dominance using an ‘alignment test’ (Rombouts et al., 1996). The participant watched an embedded video of the instructions and read the written instructions. The participants then made a small triangle with the thumb and forefinger of both hands, framing an object on a wall with the gap in the triangle. If the participant could no longer see the object through the gap in their hands when they closed their right eye, they were deemed right-eye dominant. Otherwise, they were categorised as left eye dominant (Rombouts et al., 1996). CS and VA were measured with and without monocular blur to confirm the effect of the lens on the participants’ vision.

The video clips used in the heading perception task were generated in Vizard 5 (version 5.9, WorldViz, Santa Barbara, CA). The experiment was built using Gorilla Task Builder 1 and hosted on Gorilla Experiment Builder (https://app.gorilla.sc [Gorilla Experiment Builder, 2022]) between December 2021 and February 2022.

### Materials and Stimuli

Participants reported their gender, height, weight, highest educational level, and hand with which they were using their computer mouse. They then reported any relevant medical or visual condition and whether they required a carer.

**Figure 1.**
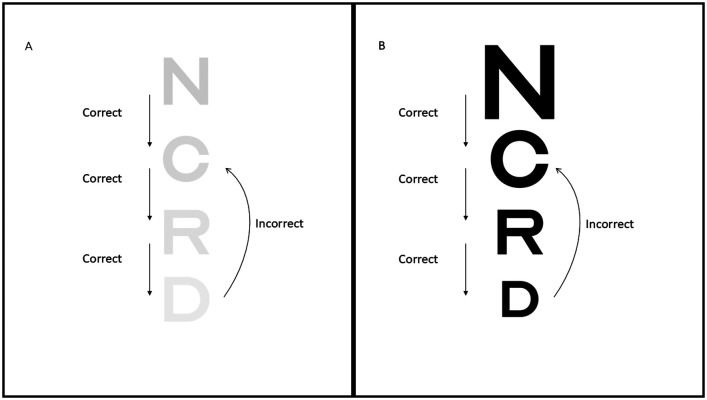
(A) Example of the varying contrast of the letters in the CS task, whereby the letters get paler with each correct answer and darker with each incorrect answer. (B) Example of the varying size of the letters in the VA task, whereby the letters get smaller with each correct answer and larger with each incorrect answer.

CS was measured by presenting participants with letters of varying contrast on-screen (starting at 10% and ranging between 1% and 100%). If participants answered correctly, the following letter presented was of 1% lower contrast than the previous letter. If the participant answered incorrectly, the following letter was of 2% higher contrast than the previous letter; see Figure 1A for an example. The participants identified 40 letters (4 × 10 [C, D, H, K, N, O, R, S, U, V]). The participants sat at arm's length from the screen and entered their answers using the keyboard. The size of the letters was 0.5 logMAR.

VA was measured at 3 m using a digital version of a logMAR chart. Letters of varying sizes were presented (starting at 1.0 logMAR, with a minimum of −0.2 logMAR), with a correct answer reducing the size of the subsequent letter by 0.1 logMAR and an incorrect answer increasing the size of the subsequent letter by 0.2 logMAR, see Figure 1B for an example. A score of −0.2 represents good VA, and 1.0 represents poor VA (Bailey & Lovie, 1976). The participants identified 40 letters (4 × 10 [C, D, H, K, N, O, R, S, U, V]). The researcher entered the participant's answers using the keyboard.

**Figure 2.**
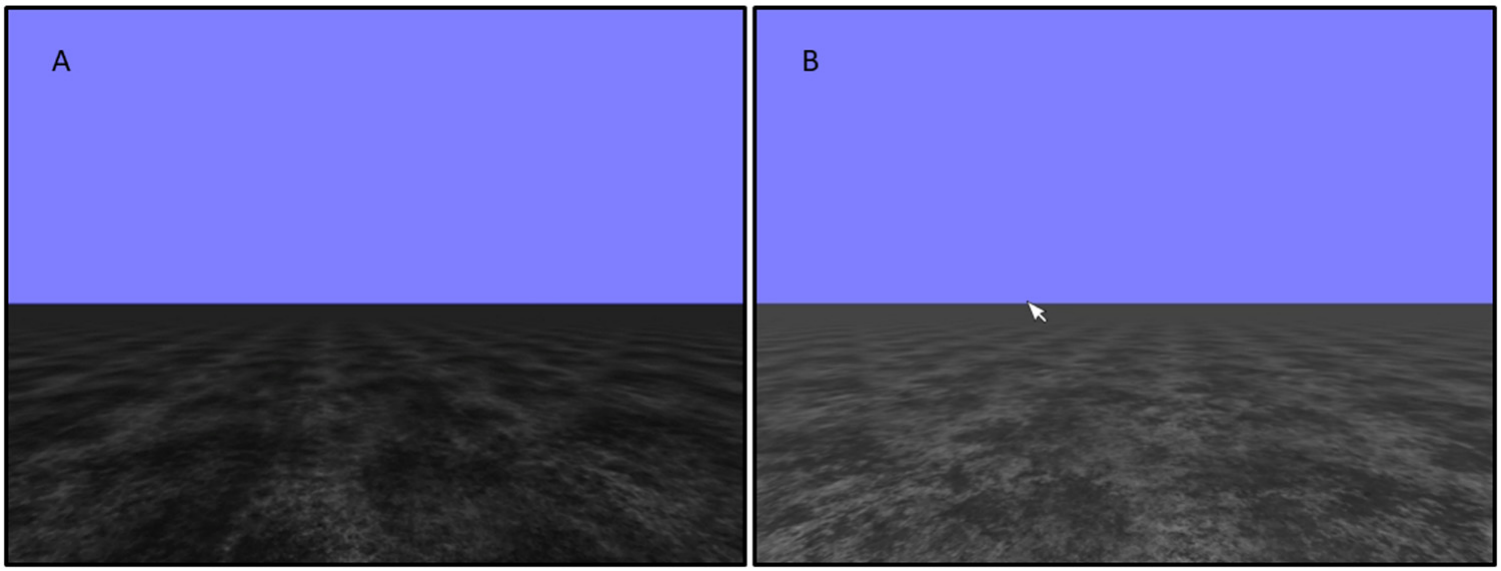
(A) A representation of the moving scene as seen by participants in the heading perception task. (B) The participants viewed a static scene at the end of each trial; they then clicked the point on the horizon toward which they were heading.

Heading perception was measured using a novel task, whereby participants watched a 3 second video clip of a computer-generated moving textured grey ground plane under a blue skyscape (Figure 2A). Upon completion, a still image of the last frame remained on the screen (Figure 2B). The participant was then required to use the left computer mouse button or trackpad to click on the point on the horizon towards which they were heading. Where the participant clicked on the screen was not controlled; however, the participants generally conformed to this instruction (horizon position = 255 pixels (px) on the *y*-axis, mean click position = 255.24px, *SD* = 6.21px). Participants sat at arm's length from the screen, and the videos took up 12° of visual field in the horizontal plane with a 4:3 aspect ratio. Heading offsets were varied in 4° steps between −20 (left) and +20 (right) degrees (0°, 4°, 8°, 12°, 16° and 20° to the left and right). The researchers manipulated the quality of the flow field signal by changing the contrast of the ground plane: ‘full flow’ (0% contrast reduction), ‘slightly degraded’ (50% contrast reduction), and ‘highly degraded’ (90% contrast reduction).

Before completing the main heading perception task, the participants completed two practise tasks—stationary and moving (in that order). Participants saw a stationary image of the ground plane, similar to those shown in Figure 2, with a red cross superimposed on the horizon. They were then required to click a ‘Continue’ button with the mouse at the bottom of the screen. The red cross then disappeared, and the participant had to click on the point on the horizon where the cross had been. The participants repeated this sequence five times, with the cross moving to different locations on the horizon. This control task served to ensure that the participant was able to see and respond accurately to the visual stimulus in the absence of any motion signal. The participants then completed the moving practice task. The moving practice task followed the same procedure as the main task; however, the angle of the movement of the ground plane was offset by 40°. These tasks allowed the participants to become accustomed to how the experiment would run and set the expectation that they were to click on the horizon in each trial. Data from these tasks were also used for data cleaning (described below).

### Statistical Analysis

Due to the repeated measures design, i.e., the same participants performing the same task under different conditions, a statistical dependency will likely exist between observations. Rather than using a traditional regression model, the researchers took a multilevel approach to correct for these dependencies (Maas & Snijders, 2003).

Data cleaning and pre-processing were carried out using the following procedure: Firstly, absolute heading error (degrees) was calculated as the angular difference between the simulated direction of heading and the judged direction of heading. Data were excluded from further analysis if the mean heading error was greater than two standard deviations above the mean during both visual conditions for one or both practice tasks (*N*[participants] = 2). The decision to remove participants was based on performance in both visual conditions rather than in the full vision condition only. There is evidence in the data that performance improved with practice, i.e., the performance on the second visual condition improved relative to the participant's performance on the first condition. Therefore, the decision to exclude a participant was based on their performance under both conditions to minimise the influence of order effects. Participants who self-reported ocular comorbidities (*N* = 4) were also excluded from the analysis (as it would not be possible to determine whether performance deficits were due to the experimental manipulation alone or due to an interaction with their comorbidity).

For the multilevel analysis of heading perception (absolute error), the independent variables were coded and entered into the fixed effects portion of the model in a way designed to best probe the hypotheses of the study:

H1. Monocular blur increases error in heading judgements—the main effect of visual condition (monocular blur vs. full vision).
H2. Degrading the contrast of the ground plane and thus degrading the flow field increases error in heading perception judgements—the main effect of flow (full flow vs. degraded flow [collapsed across heavily degraded flow and slightly degraded flow] and heavily degraded flow vs. slightly degraded flow).
H3. The difference in heading error between the two visual conditions increases as the flow is degraded—the visual condition × flow interaction.

The model also included each participant's age (standardised), CS, and VA thresholds (standardised) for each visual condition, and the computer ID for the individual who collected the data. Each participant was allocated an independent intercept, and each participant's slope was allowed to vary between conditions. Due to the potential impact of the three different computers’ data-collection devices on performance, an independent intercept was allocated to each computer.

For the estimation of directional bias due to the visual condition and optic flow manipulations, the participant's innate bias was first estimated at a heading offset of 0° in the full vision and full flow condition. This value was used to normalise the remaining data so that the participant had a bias of 0° in this condition. A negative value represented an undershoot, i.e., the participants aiming to the right of a left-sided target, or the left of a right-sided target. The independent variables were coded in the same way as the absolute error analysis. However, due to the need to test the magnitude of the effect of visual condition and flow across the heading offsets, instead of testing the two-way visual condition × flow interaction only, the three-way visual condition × flow × absolute heading offset interaction was tested. In the case of a significant three-way interaction, we will report the main effects and the three-way interaction only. Furthermore, due to the directional nature of the analysis, the direction of the flow field (left or right) was added as a covariate. Random intercepts were allocated to each participant, computer, and direction, whilst the slope associated with the visual condition was allowed to vary within each participant.

Visual condition and participant age (standardised) were entered as fixed factors for the CS and VA models. Random intercepts were allocated to each participant and each computer. Each participant only had two outcome data points, the CS or VA threshold, under each visual condition. Therefore, there was not enough data to compute random slopes for the visual condition.

The VA data were normally distributed in all conditions and modelled using a Gaussian distribution. The CS and heading perception data were not normally distributed, so they were modelled using Inverse Gaussian and Gamma distributions. The performance of each CS and heading perception model was assessed by comparing their BICs, where a low BIC indicates a better fit, favouring models with lower numbers of factors. A BIC difference greater than 10 gives ‘very strong’ evidence favouring the model with the lower BIC value (Bauldry, 2015; Raftery, 1995). CS and heading perception had a lower BIC when modelled with the Gamma distribution than the Inverse Gaussian distribution. The model comparison data are stored in the GitHub repository (https://github.com/willsheppard9895/ofAnalysis) in the files CS (/csDistTable.html), VA (/vaDistTable.html), absolute heading error (/hpDistTable.html), and directional heading error (/hpBiasTable.html).

The Inverse Gaussian and Gamma distributions only accept non-zero, positive outcome values. As the absolute heading perception data contained zeros, the data were transformed by adding one to each value. The directional heading data also contained negative values, so the data had to be transformed by adding 66 for all of the values to be positive. Transforming the data increased the intercept by one whilst leaving the slope coefficients unaffected.

For each random effect, the heterogeneity of the effect was assessed by comparing the relative size of the random and fixed effects, for example, the random slope calculated for the visual condition and the fixed effect of the visual condition. In the present case, this took the form *σ*/*β*, where *σ* is equal to the magnitude of the random effect, and *β* is equal to the magnitude of the fixed effect. When this value exceeds 0.25, we concluded that the data are heterogeneous, as a participant at the 2.5th percentile would have a score equivalent to 0.5 of the mean, and a participant at the 97.5th percentile would have a score 1.5 times the mean (Bolger et al., 2019). These results were only reported if the effect was heterogeneous.

See the analysis code and data available at (https://github.com/willsheppard9895/ofAnalysis) for details of how these models were calculated. All analyses were performed using R Statistical Software (v4.2.2, R Core Team, 2021). Generalized linear mixed models (GLMMs) were estimated using the lme4 package (Bates et al., 2015), and *p*-values were estimated using Satterthwaite's approximation through the lmerTest package (Kuznetsova et al., 2017).

## Results

### Visual Measures

#### Contrast Sensitivity

**Figure 3.**
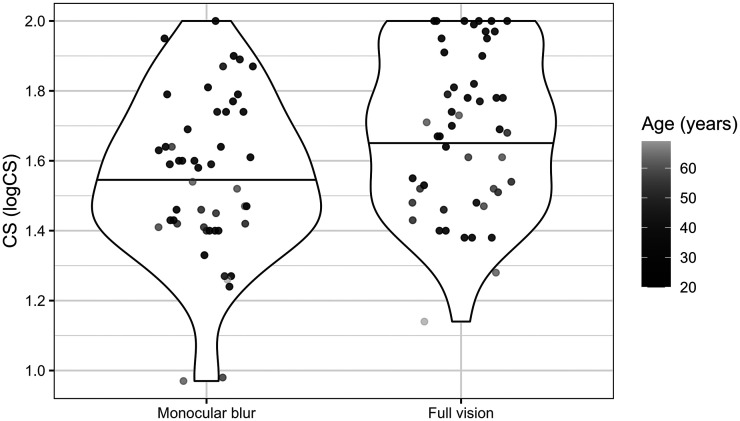
Violin plots showing CS scores by visual condition and age, where the horizontal black line represents the mean.

The *β* values presented in Table 1 predict the mean CS, *µ*_CS_. The intercept, *β*_0CS_, can be considered the predicted CS in the full vision condition for a participant of mean age with the data collected by Computer 1 and is estimated as 1.80 log units. *β*_CSmb_, the effect of visual condition on CS, predicts that monocular blur will reduce CS by 0.13 log units, as shown in Figure 3. A one standard deviation increase in age was associated with a 0.06 log unit decrease in CS. Computers two and three were associated with a 0.29 and 0.04 log unit reduction in CS relative to computer one.

**Table 1.** MLM estimates of the fixed and random effects of the visual condition, age, and computer on CS.

Fixed effects						Random effects
Parameter	Description	Mean	Upper	Lower	*p*	*σ*
*β*_0CS_	*µ*_CS_ intercept	1.80	1.89	1.71	***	0.11
*β*_CSmb_	The effect of monocular blur (mb) on *µ*_CS_	−0.13	−0.10	−0.16	***	
*β*_CSage.s_	The effect of age (standardised) on *µ*_CS_	−0.06	−0.00	−0.13	*	
*β*_CSres2_	The effect of Computer 2 on *µ*_CS_	−0.29	−0.15	−0.44	***	
*β*_CSres3_	The effect of Computer 3 on *µ*_CS_	−0.04	0.10	−0.17	***	

*p* < .05 (*), *p* < .001 (***).

#### Visual Acuity

**Figure 4.**
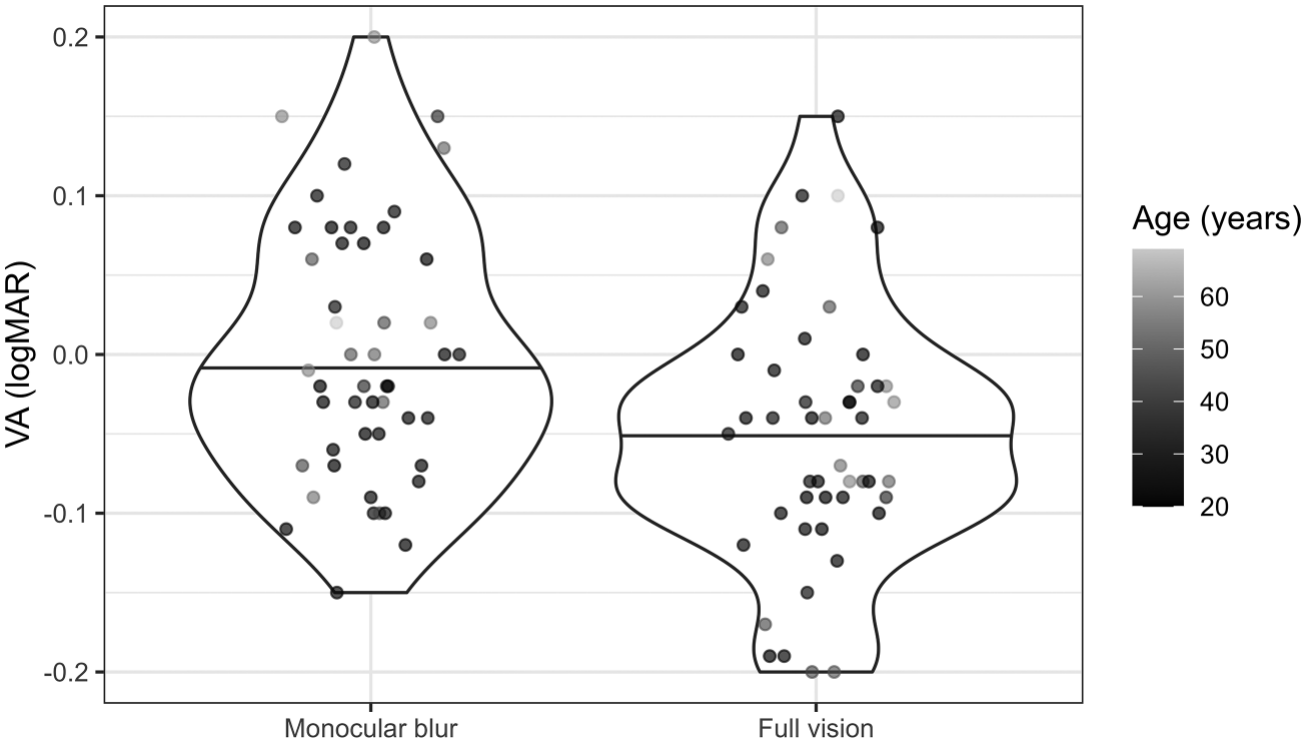
Violin plots showing VA scores by visual condition and age, where the horizontal black lines represent the mean.

The *β* values in Table 2 predict the VA thresholds, *µ*_VA_. The intercept, *β*_0VA_, can be considered the predicted VA in the full vision condition for a participant of mean age with the data collected by Computer 1 and is estimated as −0.05 logMAR. VA can be considered heterogeneous across participants as the random effect *σ*_0VA_ equals 0.06 logMAR, which exceeds 25% of the intercept. *β*_VAmb_ predicts that monocular blur is associated with a 0.05 logMAR increase in VA. Age (as per Figure 4) and the computer on which the data were collected were not associated with any significant change in VA.

**Table 2.** MLM estimates of the fixed and random effects of the visual condition, age, and computer on VA.

Fixed effects						Random effects
Parameter	Description	Mean	Upper	Lower	*p*	*σ*
*β*_0VA_	*µ*_va_ intercept	−0.05	−0.02	−0.09	**	0.06
*β*_VAmb_	The effect of monocular blur (mb) on *µ*_va_	0.05	0.07	0.03	***	
*β*_VAage.s_	The effect of age (standardised) on *µ*_VA_	0.01	0.03	−0.01	NS	
*β*_VAres2_	The effect of Computer 2 on *µ*_VA_	0.02	0.07	−0.03	NS	
*β*_VAres3_	The effect of Computer 3 on *µ*_VA_	0.00	0.05	−0.05	NS	

*p* > .05 (NS), *p* < .01 (**), *p* < .001 (***).

### Heading Perception

#### Absolute Heading Error

The *β* values presented in Table 3 predict the absolute heading error, *µ*_HP_. The intercept, *β*_0HP_, has no meaningful interpretation due to the flow level variable's coding structure. The random effect standard deviation, *σ*_0HP_, was equal to 1.46°.

**Table 3.** MLM estimates for the fixed effects of the visual condition and flow level on absolute heading error.

Fixed effects						Random effects
Parameter	Description	Mean	Upper	Lower	*p*	*σ*
*β*_0HP_	*µ*_HP_ intercept	10.24	14.01	6.47	***	1.46
Main effects						
* β*_HPmb_	The effect of monocular blur (mb) on *µ*_HP_	0.62	1.19	0.05	*	1.01
* β*_HPdf_	The effect of degraded flow (df) relative to full flow on *µ*_HP_	4.41	4.60	4.22	***	
* β*_HPhdf_	The effect of highly degraded (hdf) flow relative to slightly degraded flow on *µ*_HP_	7.17	7.49	6.85	***	
Two-way interaction effects						
* β*_HPmb*df_	The interaction effect of monocular blur × degraded flow relative to full flow on *µ*_HP_	1.18	1.56	0.80	***	
* β*_HPmb*hdf_	The interaction effect of monocular blur × highly degraded flow relative to slightly degraded flow on *µ*_HP_	2.15	2.78	1.51	***	
Covariates						
* β*_HPage.s_	The effect of age (standardised) on *µ*_HP_	−0.32	0.33	−0.97	NS	
* β*_HPcs.s_	The effect of CS (standardised) on *µ*_HP_	−0.38	0.28	−1.05	NS	
* β*_HPva.s_	The effect of VA (standardised) on *µ*_HP_	0.00	0.40	−0.39	NS	
* β*_HPres2_	The effect of Computer 2 on *µ*_HP_	0.00	1.68	−1.68	NS	
* β*_HPres3_	The effect of Computer 3 on *µ*_HP_	1.13	2.59	−0.34	NS	

All heading error values had one added to them to make them positive. Adding one to all heading error values will increase the displayed intercept value by one but will not affect the other coefficient estimates. Regarding random effects, each participant was allowed a unique intercept, and the slope associated with each visual condition was allowed to vary between participants. *p* > .05 (NS), *p* < .05 (*) , *p* < .001 (***).

There was a main effect of the visual condition, *β*_HPmb_, whereby monocular blur increased heading error by 0.62°. It should be noted that performance was heterogeneous across participants, *σ*_HPmb_ = 1.01°, with 25% of participants performing no better with full vision.

Degrading optic flow, *β*_HPdf_, increased heading error by 4.41° compared with the full flow condition. When comparing the effects of slightly degraded and highly degraded flow, *β*_HPhdf_, highly degraded flow increased heading error by 7.71°.

**Figure 5.**
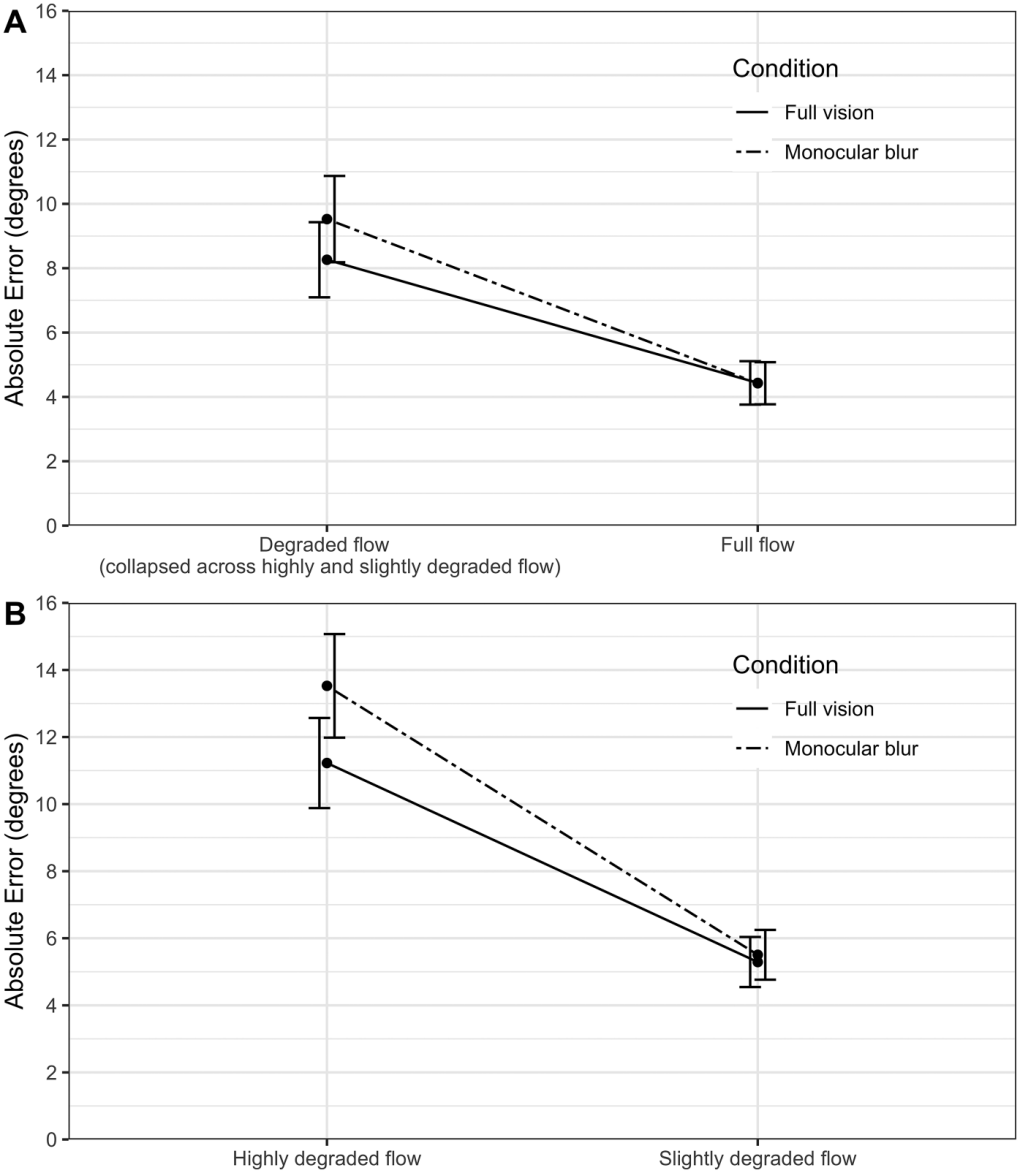
A. Absolute (unsigned) heading error across visual conditions (full vision vs. blur) and optic flow conditions (full flow vs. degraded flow). Error bars show standard error (*SE*). B. Absolute (unsigned) heading error across visual conditions (full vision vs. monocular blur) and optic flow conditions (slightly degraded flow vs. heavily degraded flow). Error bars show *SE*.

There was also a visual condition × degraded flow interaction. The difference in heading error between the visual conditions, *β*_HPmb_ _*_ _df_, was 1.18° higher when the flow was degraded, see Figure 5A. Furthermore, the difference between the visual conditions, *β*_HPmb_ _*_ _hdf_, was 2.15° higher when the flow was highly degraded than slightly degraded, see Figure 5B.

#### Directional Heading Error (Bias)

The *β* values presented in Table 4 predict the directional heading error (bias), *µ*_B_. The intercept, β_0HP_, has no meaningful interpretation due to the flow level variable's coding structure. The random effect standard deviation, *σ_0B_*, was equal to 0.92°.

**Table 4.** MLM estimates for the fixed effects of the visual condition, flow level, and heading offset on directional heading error (bias).

Fixed effects						Random effects
Parameter	Description	Mean	Upper	Lower	*p*	*σ*
* β*_0HP_	*µ*_HP_ intercept	71.88	76.36	67.40	***	0.92
Main effects						
* β*_Bmb_	The effect of monocular blur (mb) on *µ*_B_	−1.14	−0.32	−1.97	**	1.10
* β*_Bdf_	The effect of degraded flow (df) relative to full flow on *µ*_B_	−2.73	−2.15	−3.31	***	
* β*_Bhdf_	The effect of highly degraded flow (hdf) relative to slightly degraded flow on *µ*_B_	−5.87	−5.22	−6.51	***	
* β*_Bo.s_	The effect of offset (standardised; o) on *µ*_B_	0.92	1.14	0.70	***	
Two-way interaction effects						
* β*_Bmb*df_	The interaction effect of monocular blur × degraded flow relative to full flow on *µ*_B_	−1.54	−0.60	−2.48	**	
* β*_Bmb*hdf_	The interaction effect of monocular blur × highly degraded flow relative to slightly degraded flow on *µ*_B_	−2.86	−1.81	−3.92	***	
* β*_Bmb*o.s_	The interaction effect monocular blur × offset (standardised) on *µ*_B_	−0.53	−0.09	−0.97	*	
* β*_Bdf*o.s_	The interaction effect of degraded flow relative to full flow × offset (standardised) on *µ*_B_	−1.53	−1.06	−2.00	***	
* β*_Bhdf*o.s_	The interaction effect of highly degraded flow relative to slightly degraded flow × offset (standardised) on *µ*_B_	−3.36	−2.83	−3.89	***	
Three-way interaction effects						
* β*_Bmb*df*o.s_	The interaction effect of monocular blur × degraded flow relative to full flow × offset (standardised) on *µ*_B_	−0.70	0.24	−1.64	NS	
* β*_Bmb*hdf*o.s_	The interaction effect of monocular blur × highly degraded flow relative to slightly degraded flow × offset (standardised) on *µ*_B_	−1.45	−0.39	−2.51	**	
Covariates						
* β*_Bage.s_	The effect of age (standardised) on *µ*_B_	−1.28	−0.64	−1.92	***	
* β*_Bcs.s_	The effect of CS (standardised) on *µ*_B_	0.07	0.88	−0.74	NS	
* β*_Bva.s_	The effect of VA (standardised) on *µ*_B_	0.23	0.78	−0.32	NS	
* β*_Bres2_	The effect of Computer 2 on *µ*_B_	−1.93	−0.15	−3.70	*	
* β*_Bres3_	The effect of Computer 3 on *µ*_B_	−0.01	1.43	−1.46	NS	
* β*_Br_	The effect of heading offsets moving towards the right (r) of the screen on *µ*_B_	−2.30	−1.86	−2.74	***	

All directional heading error values had 66, the smallest integer to make them positive, added to them to make them positive. Whilst this does increase the displayed intercept values by the same value, it does not affect the other coefficient estimates. Regarding random effects, each participant was allowed a unique intercept, and the slope associated with each visual condition was allowed to vary between participants. *p* > .05 (NS), *p* < .05 (*), *p* < .01 (**), *p* < .001 (***).

There was a main effect of the visual condition, *β*_Bmb_, whereby monocular blur increased the underestimation of heading angle by 1.14°. It should be noted that performance was heterogeneous across participants, *σ*_Bmb_ = 1.10°.

Degrading optic flow, *β*_Bdf_, led to an underestimation of the heading angle by 2.73° compared with full flow. Furthermore, highly degraded flow, *β*_Bdf_, led to an underestimation of the heading angle by 6.51° compared with the slightly degraded flow.

**Figure 6.**
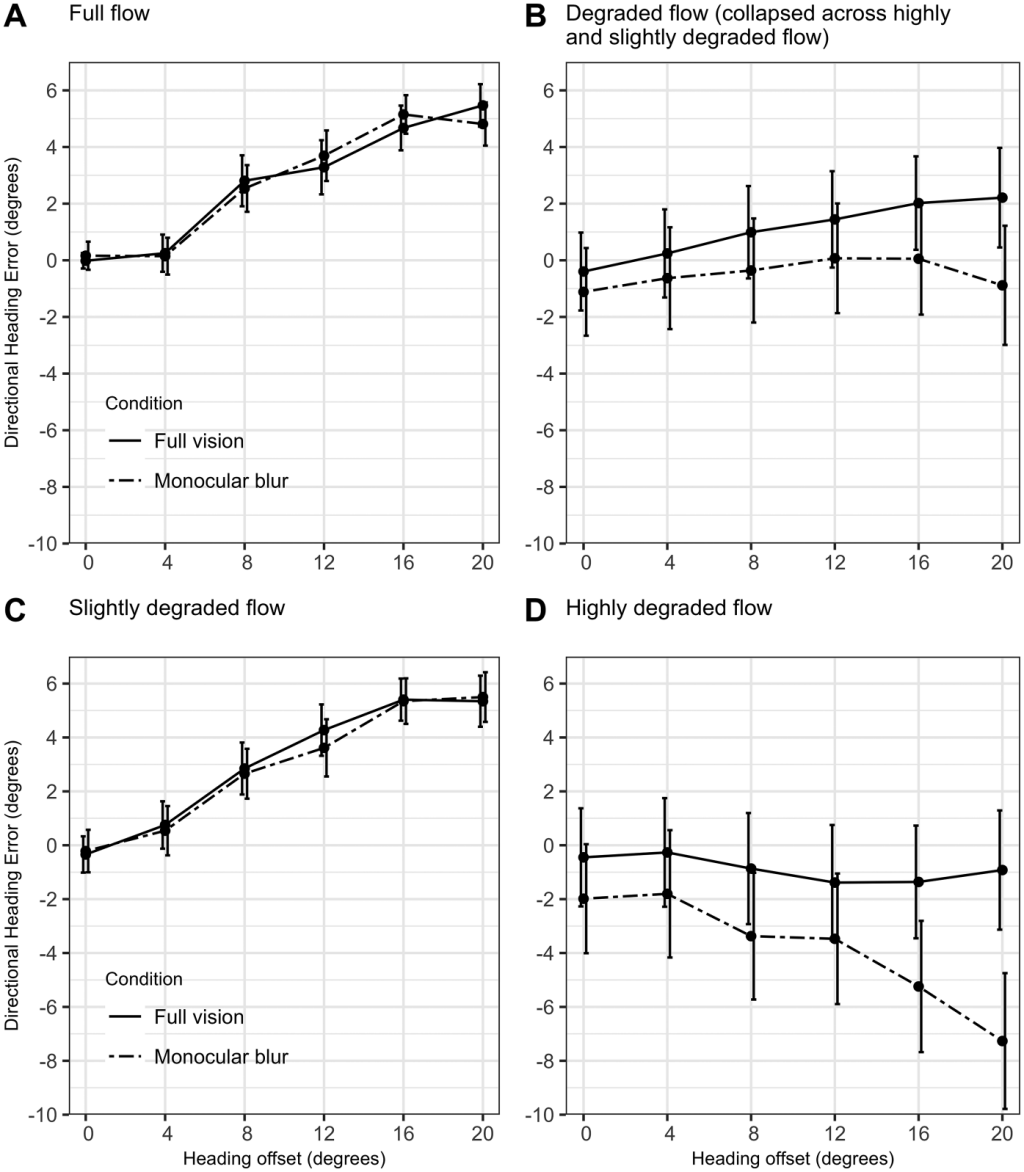
Directional heading error (bias) across visual conditions (full vision vs. monocular blur) with A. full flow. B. Degraded flow (collapsed across slightly degraded flow and highly degraded flow). C. Slightly degraded flow. D. Highly degraded flow. Error bars = *SE*.

A 1 *SD* increase in heading offset (6.79°) was associated with increased overestimation of the heading angle by 0.71°, equivalent to 10.46% of the heading angle. However, a significant three-way interaction between visual condition × highly degraded flow × offset interaction, *β*_Bmb_ _*_ _hdf_ _*_ _o.s_, indicated that this effect was not consistent across the conditions. Whereby an increase in offset was associated with more negative directional heading estimates, i.e., aiming to the right side of a left-sided target, with monocular vision and highly degraded flow only (see Figure 6D). Under all other combinations of the visual conditions and flow degradations, an increase in offset was associated with an increase in directional heading estimates, (i.e., aiming to the left of a left-sided target), or there was no relationship (see Figures 6A–C).

## Discussion

Researchers have previously shown that the presence of monocular blur impairs a person's ability to move through the world, be this on foot or when driving a car (Elliott et al., 1997, 2000; Meuleners et al., 2021; Molina et al., 2021), particularly in environments with diminished optic flow signals (Elliott et al., 1996). Optic flow is a critical perceptual signal for gauging heading, the current direction of travel (Bruggeman & Warren, 2009; Cutting et al., 1992; Gibson, 1958; Kountouriotis & Wilkie, 2013). The present study, therefore, tested three hypotheses: (i) that monocular blur increases error in heading judgements, (ii) that degrading the flow field increases error in heading judgements, and (iii) there will be an interaction between these effects, such that monocular blur will have a greater effect on the magnitude of heading error when the flow field is more degraded. The data presented in the present manuscript supported all three hypotheses after controlling for age, CS, and VA. Monocular blur was associated with a significant reduction in CS and VA (by 0.10 log units and 0.05 logMAR, respectively). However, the 0.62° increase in heading error associated with monocular blur was independent of the variance associated with CS and VA.

As expected, degrading optic flow impaired heading perception. Heavily degraded flow increased heading errors by 7.17° compared to slightly degraded flow. Degraded flow (collapsed across slightly degraded and heavily degraded flow) compared to full flow increased heading errors by 4.41°. These results confirm findings from previous research that a rich flow field is beneficial for accurate heading perception, and degrading optic flow impairs heading perception (Gibson, 1958; Koerfer & Lappe, 2020; Riddell et al., 2019), steering (Frissen & Mars, 2014; Kountouriotis et al., 2013; Nash et al., 2016; Okafuji et al., 2018), and walking (Bruggeman et al., 2007a, 2007b; Bruggeman & Warren, 2009).

When examining conditions that were both blurred and degraded, the difference in heading errors between monocular blur and full vision conditions became 1.18° larger as flow became degraded; this effect is demonstrated in Figure 5A by comparing the relative increase in heading error (seen as the distance on the *y*-axis) between the visual conditions under degraded flow relative to full flow. Furthermore, when comparing highly degraded and slightly degraded flow, the difference in heading error between the monocular blur conditions was 2.15° larger when flow was heavily degraded, again, with monocular blur associated with more error, as demonstrated as increased distance on the *y*-axis between the visual conditions under heavily degraded flow compared with slightly degraded flow (see Figure 5B). This evidence is consistent with the idea that Second-Eye cataract Surgery (SES) may be necessary to improve driving performance at night (Elliott et al., 1997, 2000), i.e., heading perception with monocular blur may be sufficient to facilitate driving during the day but not at night (when the optic flow is degraded). These findings align with previous work investigating the effect of binocular visual blur on the successful completion of an obstacle course (Elliot et al., 1996). This study simulated cataracts (compared to full vision) and found that the number of obstacles collisions only increased under low ambient illumination, with similar performance in both visual conditions when the course was fully lit (Elliott et al., 1996). The improvement in optic flow perception following SES is also consistent with the finding that drivers are less likely to avoid driving at night following SES compared to before (Agramunt et al., 2018).

Whilst the pattern of results regarding the concurrent effects of visual blur and degrading the flow field on absolute heading error is clear, the effects of these factors on directional heading errors are more nuanced. It appears that under full flow and slightly degraded flow conditions, the participants tended to overestimate the heading angle by approximately 25% to 30% of the actual heading angle (see Table 5). However, once flow becomes highly degraded, participants tend to underestimate the heading angle (see Table 5). This is particularly evident when the flow is highly degraded and the participant is exposed to monocular blur, whereby the mean underestimation of the heading angle was 37% of the actual heading angle.

**Table 5.** The mean directional heading error (bias) as a percentage of the heading offset in each visual condition across the three flow conditions [±95% CIs].

	Full vision	Monocular blur
Full flow	+25.06% [+3.56% to +46.55%]	+24.47% [+0.74% to +48.19%]
Slightly degraded flow	+30.10% [+15.78% to +44.41%]	+27.54% [+11.42% to +43.66%]
Highly degraded flow	−8.43% [−14.08% to −2.79%]	−37.06% [−50.04% to −24.07%]

A simple model of a driving situation can be created to better understand what the increase in heading error associated with monocular blur and degraded visual information could potentially mean for drivers in relation to real on-road behaviours. Taking the main effect of monocular blur (the most conservative measure of the impairment in heading perception currently presented), a vehicle with a width of 1.7 m (the most commonly registered car in the UK, Ford Fiesta, 2023 [Vehicle Licensing Statistics, 2023]) driven along the centre of a single-carriageway road in the UK (with a minimum width of 3 m [Highways Act, 1980]), gives the driver 0.65 m of lateral deviation before the vehicle begins to leave the lane. If the additional heading error due to monocular blur (0.62°) were undetected and left uncorrected, the car would begin to leave the roadway after 60.1 m of forward travel, which would take 2.24 s when travelling at 60 miles per hour (the national speed limit for a single-carriageway road in the UK; 26.8 m/s). For context, Calvi et al. (2015) measured reaction times to sudden braking events when driving on (simulated) urban roads, rural roads, and highways. When the participants were not distracted, they exhibited reaction times of 1.18 s (95% CIs = 0.71–1.65 s) on a curved urban road, and 1.83 s (95% CIs = 0.87–2.79 s) on a curved rural road. Assuming the range of reaction times would be similar when responding to heading errors, when under urban conditions, some individuals may only have a safety margin of 0.59 to 1.53 s to notice and correct the heading error before they begin to leave the road. However, when conditions are sub-optimal, such as on winding rural roads, an individual may well be too late to avoid leaving the road, with safety margins in the range of −0.55 s to 1.37 s. This safety margin stands to be reduced further in the case of sub-optimal driving conditions such as fog. Research investigating safety margins of Glaucoma patients during cornering indicated that in more adverse driving conditions (high speed and no fog vs. high speed and fog), the Glaucoma patients showed greater reductions in safety margin (less safe driving) compared to controls than they did in less adverse driving conditions (low speed and no fog vs. low speed and fog; [Diniz-Filho et al., 2016]). When considered alongside the results presented in the current study, there is clear evidence that the combination of visual deficits and degraded visual information will have a negative effect on one's ability to perform tasks such as driving. It seems that an impaired ability to use the optic flow signals may not only diminish the ability to determine one's own direction of travel but also the direction of travel of others. For example, it has been demonstrated that the parsing of optic flow signals can be used to predict the heading of other objects in the scene (Warren & Rushton, 2008a, 2008b), however, further experimental work is needed to determine exactly how blur and degraded flow affects these functions.

Whilst reaction times will vary by age and situation, it does seem likely that there will be some situations where heading errors of the magnitude observed in the present study could be sufficient to cause a vehicle collision, crash or near-miss. Nevertheless, some caution is needed to directly relate reaction times to heading judgements, with steering control. Research has shown that accurate heading perception is sometimes insufficient to produce accurate steering under the same visual conditions. For example, when participants were asked to make heading judgements and steering responses when presented with degraded flow, they demonstrated significant understeering despite being able to produce accurate heading judgements (Kountouriotis & Wilkie, 2013). These findings distinguished between heading perception and steering, suggesting that accurate steering is not necessarily a product of accurate perception of heading. To directly investigate the links between monocular blur, heading perception and steering, future research should explicitly test whether an individual's heading perception predicts their ability to detect and correct steering errors under full and degraded flow conditions. The independence of the blur effects from CS/VA might suggest that there would be merit in directly assessing an individual's heading perception when making decisions regarding their return to driving following the correction of a condition causing monocular blur, such as cataract removal. Future work must investigate the relationship between heading perception and driving ability and whether these effects are also evident in individuals with pathological monocular blur rather than artificially induced blur.

Whilst monocular blur negatively impacted heading perception for most people in our study, this effect was not universal. For 13 of the 52 participants, heading error did not increase, and in some cases decreased, in the monocular blur condition. One explanation for this deviation from the expected effect of monocular blur on heading error may be due to order effects. Ten of the 13 participants performed the full vision condition first, so they would have been well practised when they performed the heading task with monocular blur. The three remaining participants completed the monocular blur condition first: none reported visual comorbidities, and all three performed better in the CS and VA tests with full vision. These deviations from expected performance do not seem to be due to demographic differences between the groups as the *mean *ages are similar (full vision first = 27.2 years and monocular blur first = 28.0 years), and the modal highest level of education was the same for both groups (‘A’ or ‘AS’ level). The testing sessions lasted 40 to 60 min, so these participants may have been fatigued by the time they performed the full vision condition. These issues may be mitigated in future research by participants completing shorter blocks of each visual condition, repeated more often.

The effects of monocular visual blur can significantly affect an individual's life. Cataract removal provides a convenient test case for comparing functions with and without monocular blur. It is typical in parts of the UK for bilateral cataract patients to have their cataracts removed in two separate surgical sessions (Dickman et al., 2022). The gap between the surgeries leads to a period of living with monocular visual blur following the removal of the first cataract (First-Eye Surgery; FES). A clinician will assess the patient's visual function, typically using QoL or VA measures, and depending on the results, the second cataract may (or may not) be removed (Lisle et al., 2021). The transition from monocular blur (post-FES) to full vision (post-SES) is a helpful proxy for the real-world benefits of removing monocular blur, and despite some contradictory evidence from studies using self-report measures (Feng et al., 2018; Foss et al., 2006; Gia To et al., 2014), SES has been linked to objective improvements in mobility (Elliott et al., 1997, 2000; Lee et al., 2013) and a reduction in fall risk (Meuleners et al., 2014).

SES improves certain aspects of driving. For example, in a driving simulator study, SES was associated with reduced crashes/near-crashes and reduced speed limit violations (Meuleners et al., 2021). It is typical for individuals to self-regulate, limiting their driving in certain situations to compensate for reduced capabilities. After SES, the percentage of people who self-regulate for night-time driving (a situation associated with reduced optic flow information) and driving in heavy traffic (a situation that is potentially associated with a reduction in optic flow information due to the obstruction of the visual field) is reduced from 21.7% to 10.9% and 8.3% to 2.1%, respectively (Agramunt et al., 2018). Questionnaire data further support the idea that timely SES is necessary to maintain an individual's night-time driving ability (Elliott et al., 1997, 2000). Reduced mobility, increased fall risk, and driving cessation are associated with reductions in emotional, physical, and psychosocial well-being, as well as increased likelihood of entering residential care and increased mortality (Chihuri et al., 2016; Farragher et al., 2014; Olaya et al., 2018; Schoene et al., 2019). Therefore, a nuanced understanding of monocular visual blur's behavioural and perceptual impact, particularly in situations associated with reduced optic flow information, is imperative to better support the affected individuals.

To this end, we have demonstrated that monocular blur significantly impairs one's ability to make heading judgements. This effect is amplified when the quality of the incoming visual signal is impoverished, in this case, by degrading the flow field. This effect is independent of changes to CS and VA, which suggests that there may be a need to develop novel visual tests that can be delivered in the clinic or the patient's home to assess their ability to perceive motion and safely navigate the world following changes to their vision.

## Supplemental Material

sj-docx-1-ipe-10.1177_20416695251317148 - Supplemental material for Monocular blur impairs heading judgements from optic flow Supplemental material, sj-docx-1-ipe-10.1177_20416695251317148 for Monocular blur impairs heading judgements from optic flow by William E. A. Sheppard, Rachel O. Coats, Richard M. Wilkie and Rigmor C. Baraas in i-Perception
