# Supplementary material for: Monocular blur impairs heading judgements from optic flow
Source: Iperception. 2025 Feb 26;16(1):20416695251317148. doi: 10.1177/20416695251317148 (PMC11863211; doi:10.1177/20416695251317148)
Supplement: sj-docx-1-ipe-10.1177_20416695251317148 - Supplemental material for Monocular blur impairs heading judgements from optic flow [file sj-docx-1-ipe-10.1177_20416695251317148.docx]

# Supplementary Materials 1

The operating system, browser and screen size of the laptops used by the researchers for data collection.

| Researcher | Operating system | Browser | Screen size (pixels) |
| --- | --- | --- | --- |
| 1 | Windows 10 | Chrome 96.0.4664.110 | 1536x864 |
| 2 | Windows 10 | Edge 96.0.1054.53 | 1280x720 |
| 3 | Mac OS 10.15.7 | Chrome 96.0.4664.110 | 1440x900 |
